# Supplementary material for: Host dispersal relaxes selective pressures in rafting microbiomes and triggers successional changes
Source: Nat Commun. 2024 Dec 30;15:10759. doi: 10.1038/s41467-024-54954-z (PMC11685921; doi:10.1038/s41467-024-54954-z)
Supplement: Supplementary file 1 — Supplementary Information [file 41467_2024_54954_MOESM1_ESM.docx]

Supplementary Information for:

**Host dispersal relaxes selective pressures in rafting microbiomes and triggers successional changes**

William S. Pearman^1,2,3*^, Grant A. Duffy^1^, Robert O. Smith^1^, Kim I. Currie^4^, Neil J. Gemmell^2^, Sergio E. Morales^3^, Ceridwen I. Fraser^1^

* Corresponding author

[Wpearman1996@gmail.com](mailto:Wpearman1996@gmail.com)

**Supplementary Table S1** - Relative contributions of ecological processes to community assembly for rafts and non-rafts as inferred using the iCAMP framework.

|  | Homogeneous Selection | Heterogeneous Selection | Dispersal Limitation | Homogenising Dispersal | Drift |
| --- | --- | --- | --- | --- | --- |
| Raft | 30.8% | 2.9% | 36.4% | 1.6% | 28.3% |
| Non-Raft | 65.4% | 0.7% | 7.8% | 0.7% | 25.5% |

**Supplementary Table S2** - Relative contributions of ecological processes to assembly of the *Granulosicoccus* phylogenetic bin for rafts and non-rafts as inferred using the iCAMP framework.

|  | Homogeneous Selection | Heterogeneous Selection | Dispersal Limitation | Homogenising Dispersal | Drift |
| --- | --- | --- | --- | --- | --- |
| Raft | 9.1% | 0% | 88.7% | 0% | 2.2% |
| Non-Raft | 82.1% | 0% | 17.8% | 0% | 0.1% |

**Supplementary Table S3** - Coordinates and dates of raft collection

| Sample | Latitude | Longitude | Date Collected |
| --- | --- | --- | --- |
| K1 | -45.782167 | 170.990667 | 25/03/22 |
| MunF1 | -45.7955 | 171.020167 | 18/01/22 |
| MunF3 | -45.803667 | 171.123833 | 18/01/22 |
| MunF6 | -45.815833 | 171.112333 | 18/01/22 |
| K13 | -45.796733 | 171.22673 | 25/03/22 |
| Mu2 | -45.776 | 170.968 | 27/01/21 |
| MunF10 | -45.782333 | 171.1125 | 18/01/22 |
| MunF11 | -45.782333 | 171.1125 | 18/01/22 |
| MunF4 | -45.815833 | 171.112333 | 18/01/22 |
| S10 | -45.795833 | 171.101 | 29/03/21 |
| S2 | -45.787167 | 170.999833 | 29/03/21 |
| S3 | -45.773833 | 170.97 | 29/03/21 |
| S6 | -45.789167 | 171.053167 | 29/03/21 |
| S7 | -45.795833 | 171.101 | 29/03/21 |
| S8 | -45.795833 | 171.101 | 29/03/21 |
| S9 | -45.795833 | 171.101 | 29/03/21 |
| Mu6 | -45.801333 | 171.169333 | 27/01/21 |
| MunP1 | -45.81 | 171.249667 | 30/11/21 |
| Mu5 | -45.792 | 171.0415 | 27/01/21 |
| MunF9 | -45.782333 | 171.1125 | 18/01/22 |
| S4 | -45.789167 | 171.053167 | 29/03/21 |
| S5 | -45.789167 | 171.053167 | 29/03/21 |
| K5 | -45.807167 | 171.1325 | 25/03/22 |
| K4 | -45.807167 | 171.1325 | 25/03/22 |
| K11 | -45.8085 | 171.243167 | 25/03/22 |
| K2 | -45.782167 | 170.990667 | 25/03/22 |
| K3 | -45.807167 | 171.1325 | 25/03/22 |
| K6 | -45.806167 | 171.217 | 25/03/22 |
| MunF8 | -45.782333 | 171.1125 | 18/01/22 |
| S11 | -45.796167 | 171.1255 | 29/03/21 |
| MunF7 | -45.782333 | 171.1125 | 18/01/22 |
| MunP2 | -45.826 | 171.479 | 30/11/21 |
| Mu3 | -45.801333 | 171.169333 | 27/01/21 |
| K7 | -45.806167 | 171.217 | 25/03/22 |
| MunF2 | -45.803667 | 171.123833 | 18/01/22 |
| K12 | -45.8085 | 171.243167 | 25/03/22 |
| K8 | -45.806167 | 171.217 | 25/03/22 |

**Supplementary Table S4.** Drift properties used for particle release. Values from (74,75) via the OpenDrift Leeway model (<https://opendrift.github.io>; 66)

| **Person-in-water** |  | PIW-1 | PIW-5 | PIW-6 |
| --- | --- | --- | --- | --- |
|  |  | Unknown state  (mean values) | Scuba suit  (face up) | Deceased  (face down) |
| **Downwind** | Slope (%) | 0.96 | 0.63 | 1.117 |
|  | Offset (cm s-1) | 0 | 0 | 10.2 |
|  | Std. dev. (cm s-1) | 12 | 5.3 | 3.04 |
| **Right** | Slope (%) | 0.54 | 0.31 | 0.04 |
|  | Offset (cm s-1) | 0 | 0 | 3.9 |
|  | Std. dev. (cm s-1) | 9.4 | 4.5 | 4.05 |
| **Left** | Slope (%)% | -0.54 | -0.31 | -0.04 |
|  | Offset (cm s-1) | 0 | 0 | -3.9 |
|  | Std. dev. (cm s-1) | 9.4 | 4.5 | 4.05 |


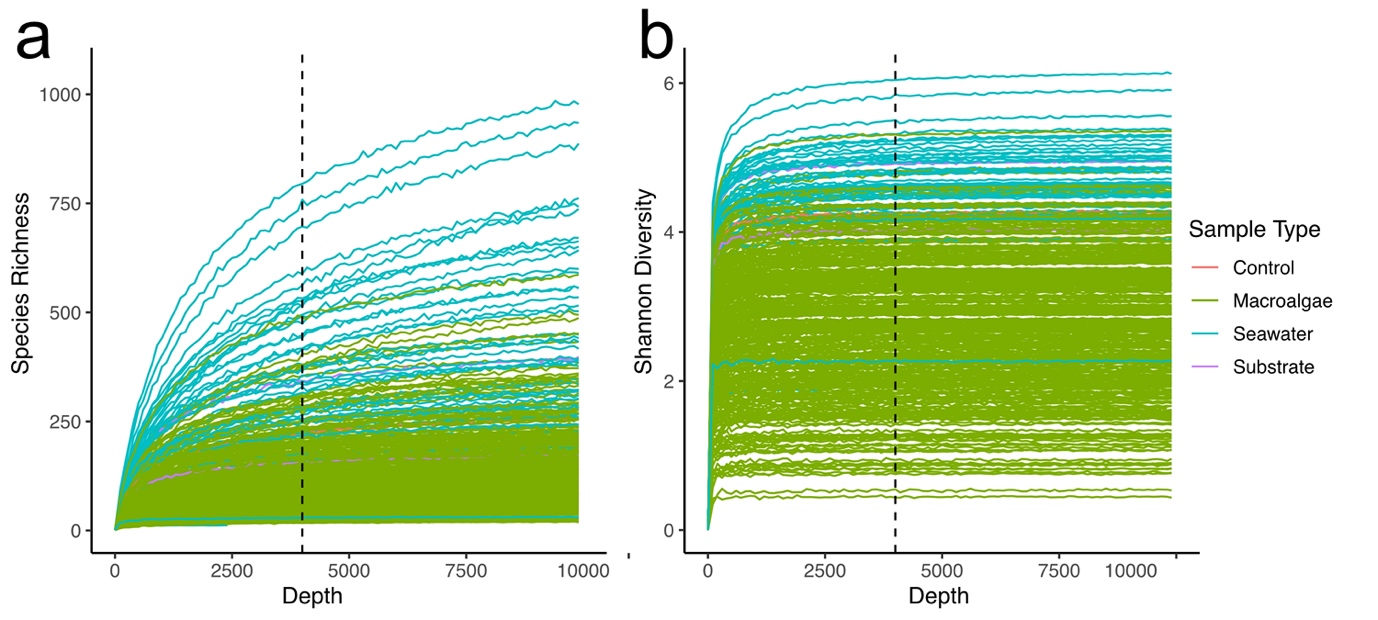


**Supplementary Figure S1** – Rarefaction curves based on mean observed richness (a) or shannon diversity (b). Dashed line indicates the value to which we rarefied data.


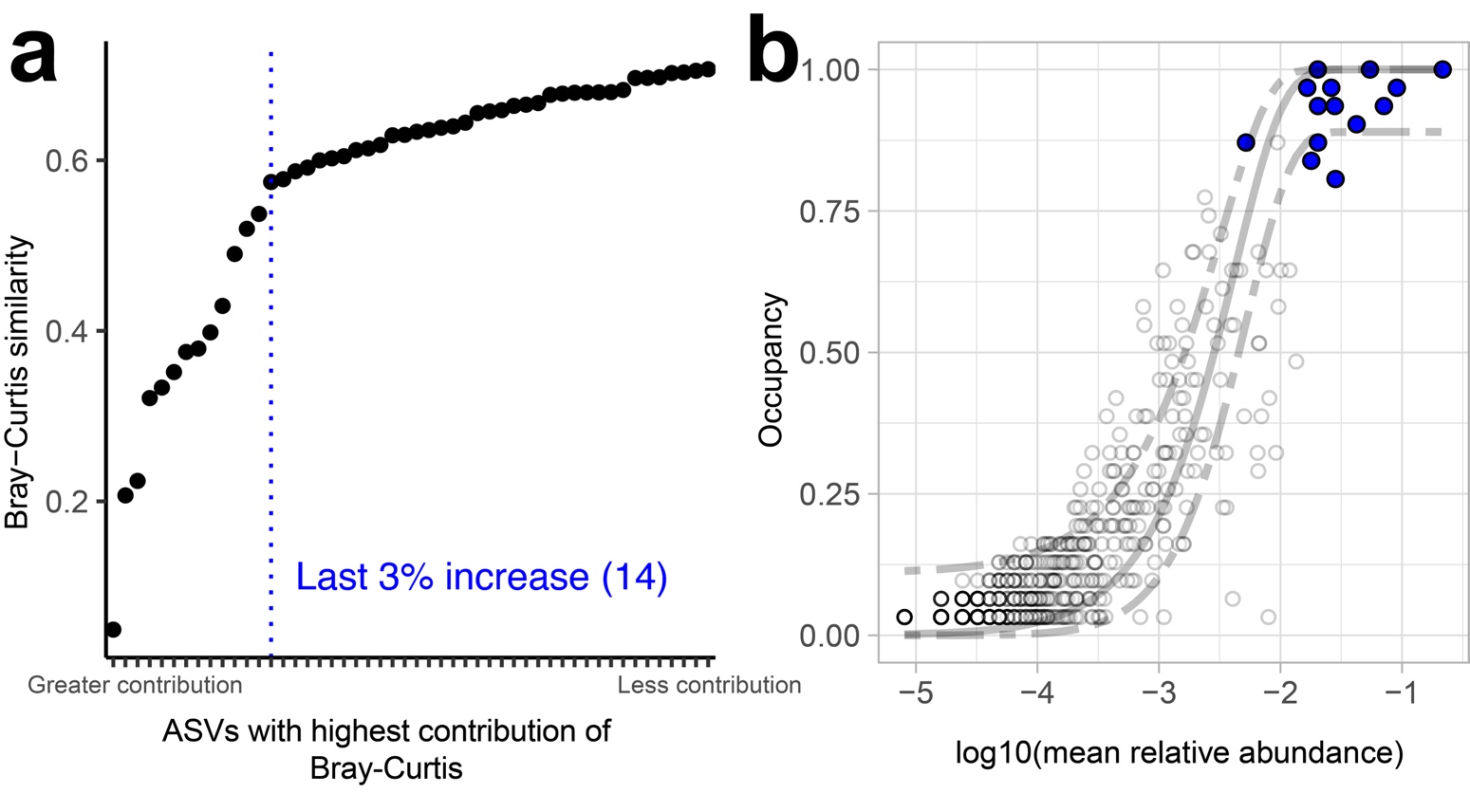


**Supplementary Figure S2** – Rank-occupancy relationships for ASVs in non-raft microbiomes. a) ASVs sorted and ranked by their contribution to overall Bray-Curtis similarity. Vertical line indicates cut-off to be considered within the ‘core’ community. b) Sloan neutral community model of ASVs, blue dots represent core microbes.


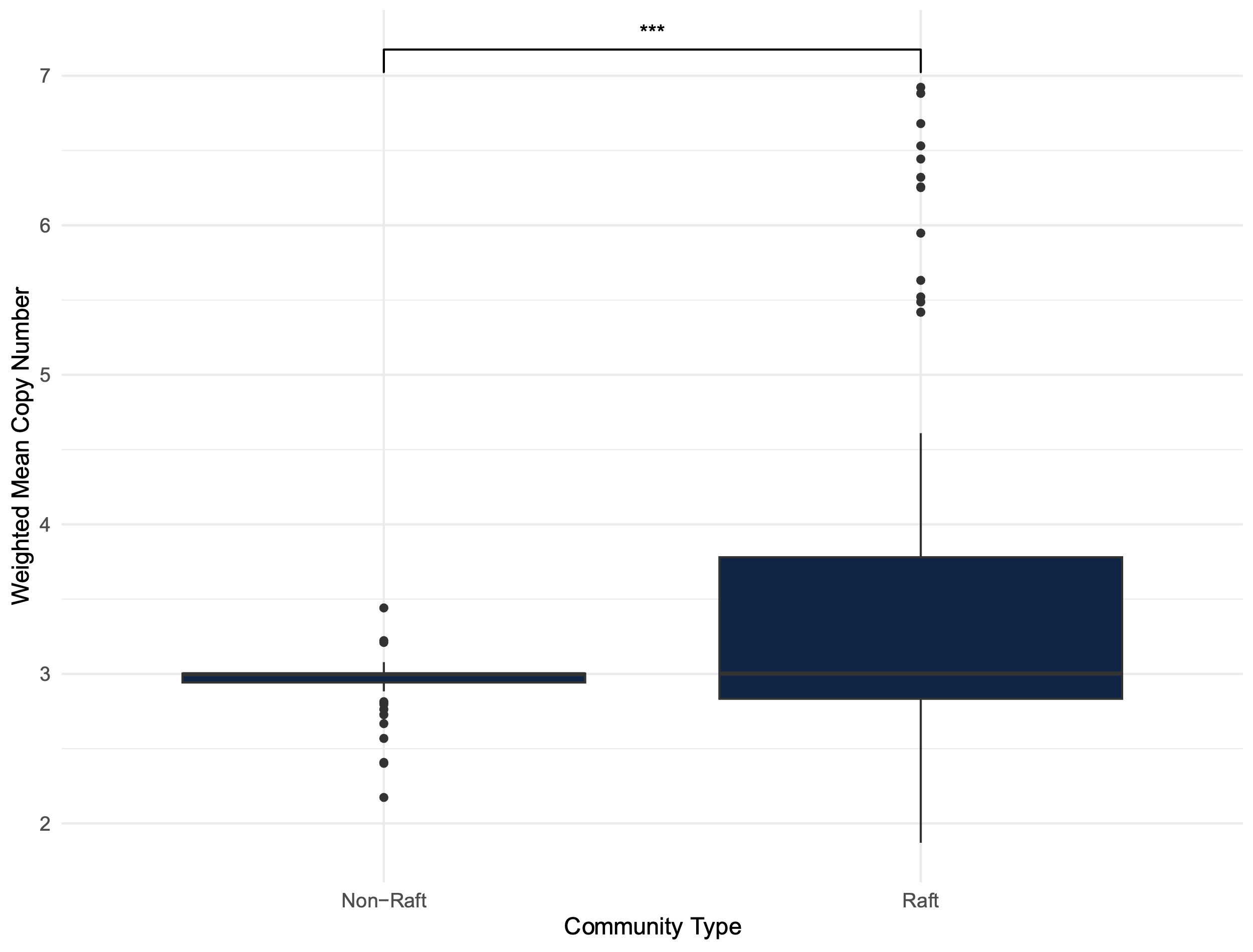


**Supplementary Figure S3** - Weighted mean copy number of rRNA operons at the genus level for rafts and non-rafts. This value is a proxy measure for the maximum growth rate of a community. *** = p < 0.001 assessed via two-sided T test (exact p-value of 1.9 x 10^-5^). N=81 and 97 for non-raft and raft samples respectively.

**
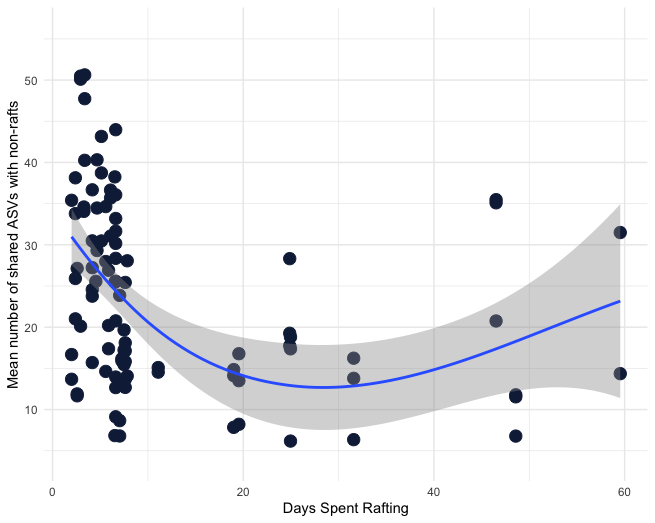
**

**Supplementary Figure S4** - Relationship between the mean number of ASVs shared between raft samples and non-raft samples as a function of raft time. Smoothed line represents GAM smoothing. N=127 microbial samples.

#
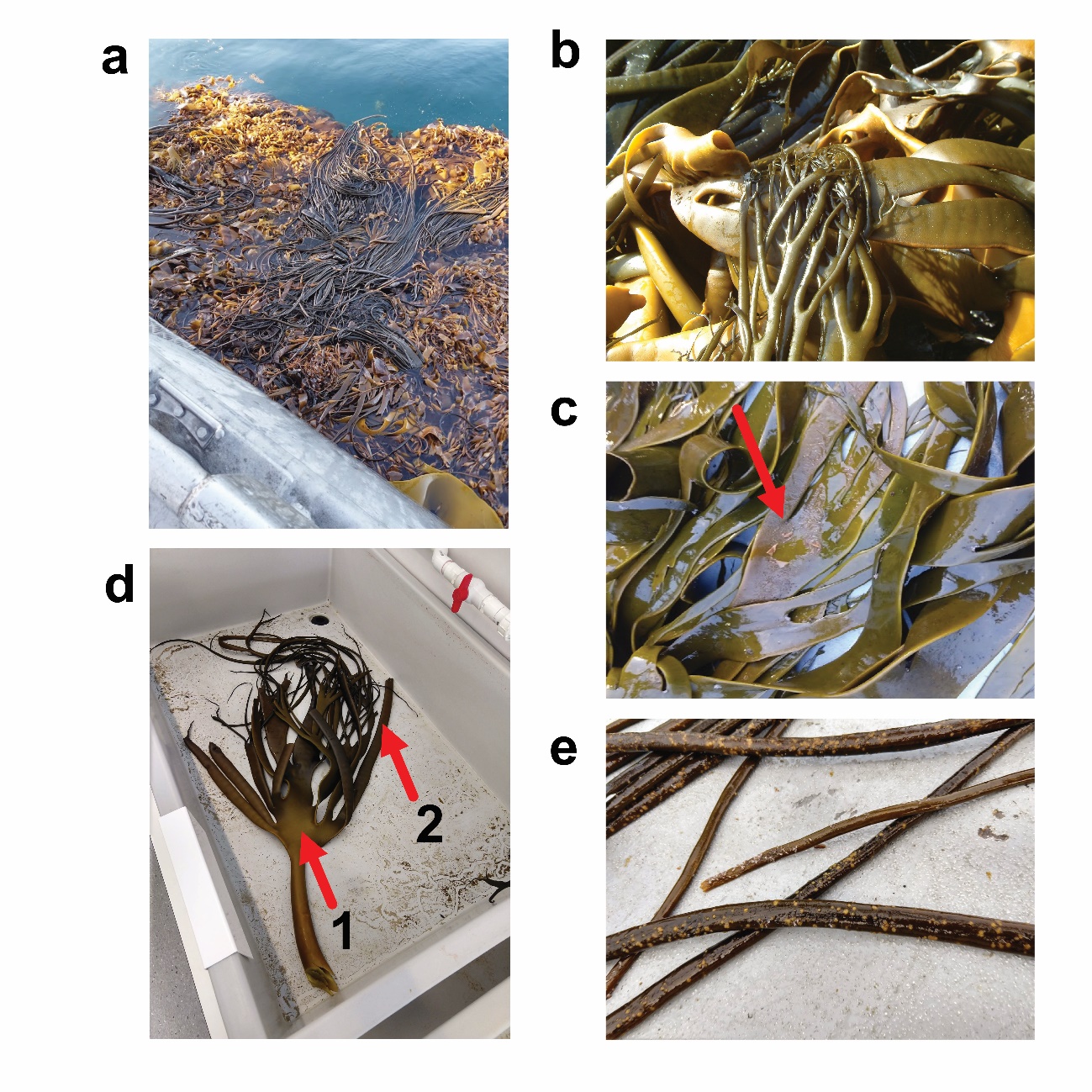


**Supplementary Figure S5** – Images of a kelp raft (a), healthy kelp (b), and unhealthy kelp rafts (c and e). In c, sloughing tissue layer is indicated by red arrow, and wart like blisters in d are indicated by a red arrow. Healthy non-raft kelp (d), red arrows indicate palmate meristem (1) and blade tissue (2) to demonstrate microbial sampling regions.

#
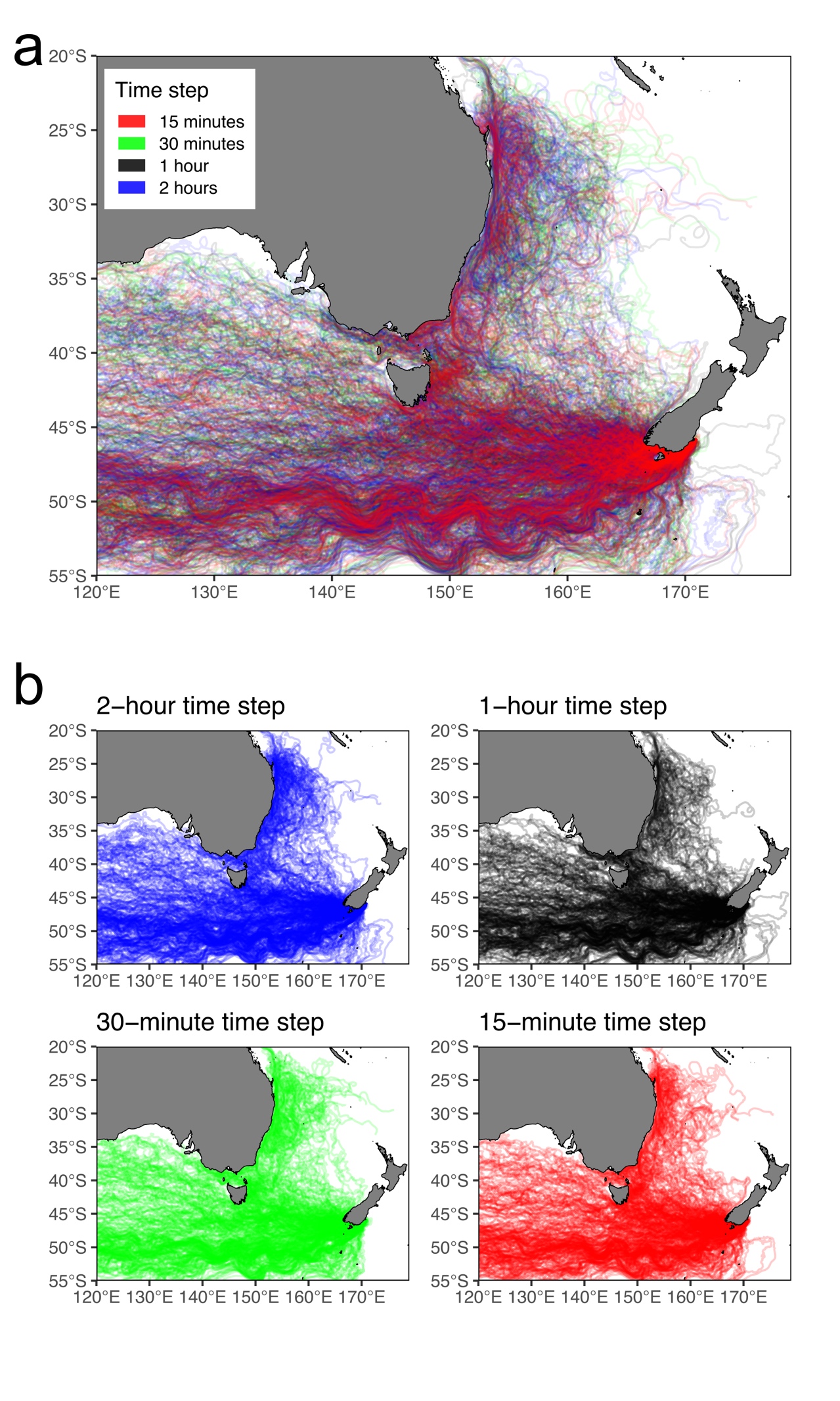


**Supplementary Figure S6** – Particle trajectories for a random raft (raft Mu2) using a range of different advection time steps within Open Drift, n=999 particles for each timestep.

*
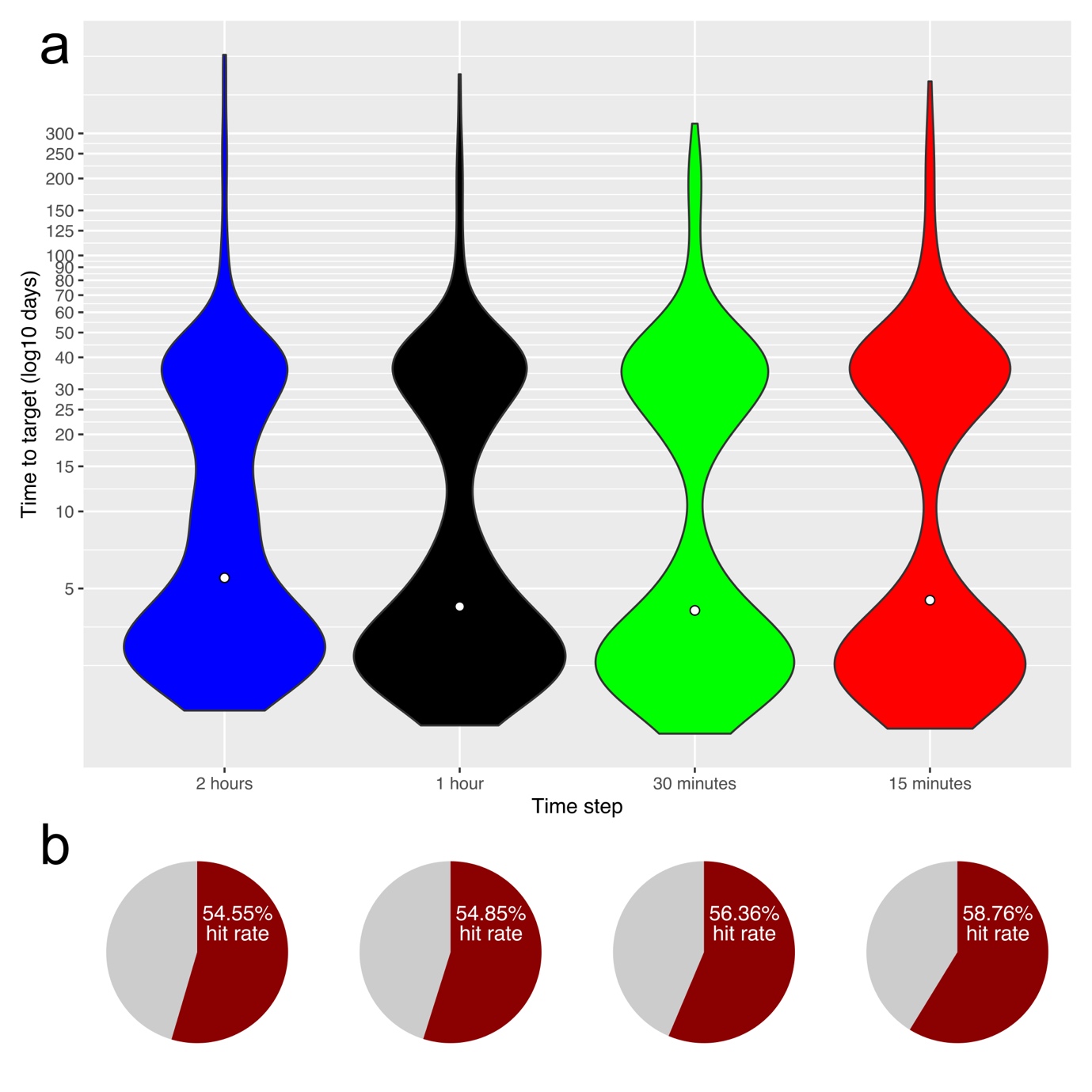
*

**Supplementary Figure S7** – a) Distributions of time to target (i.e., source location inferred via genetic analysis of raft) for raft Mu2 across a range of advection time steps in Open Drift, and b) proportion of particles reaching target/source location. 999 particles were released for each timestep

#
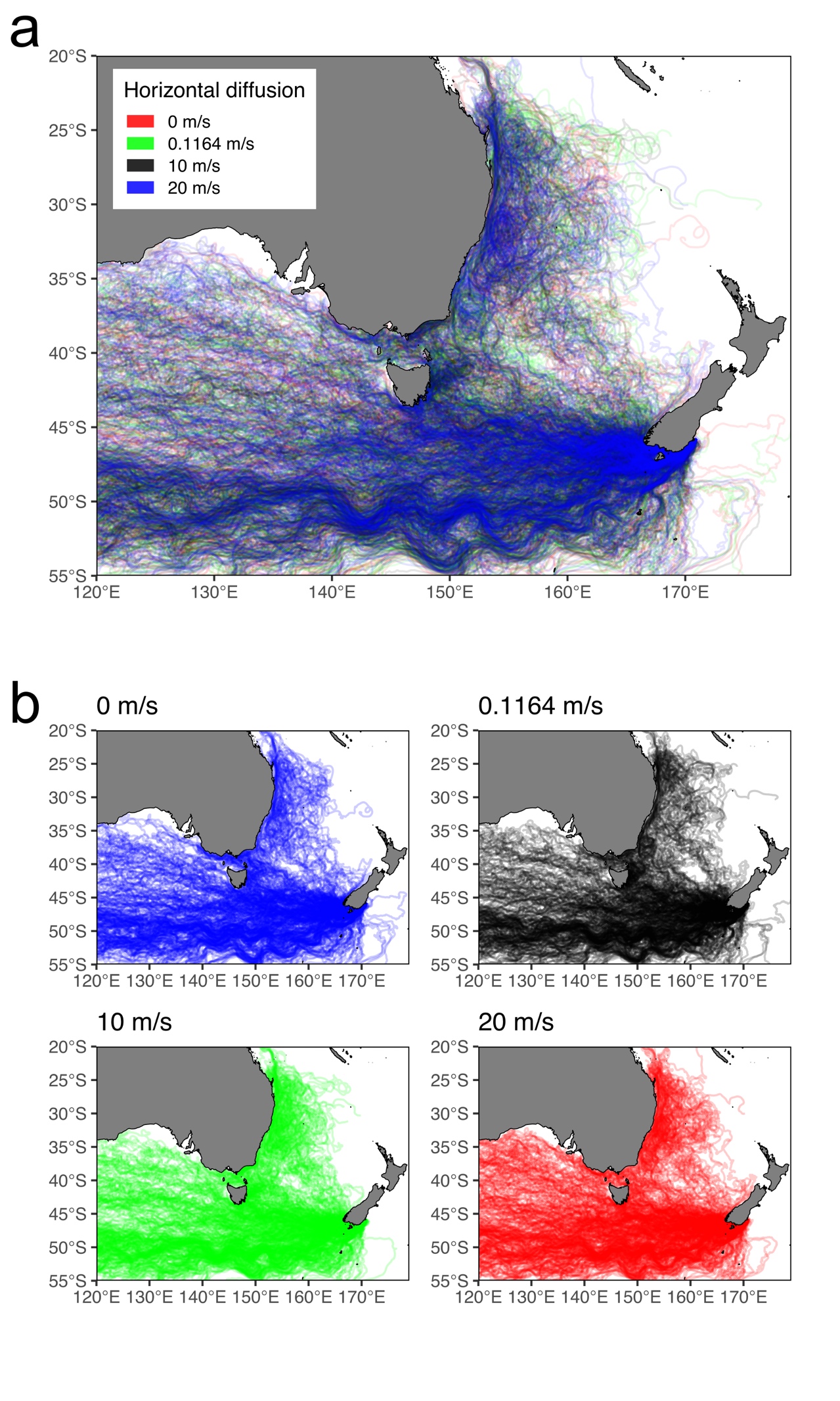


**Supplementary Figure S8** – Effects of use of different horizontal diffusivity coefficients within Open Drift on particle trajectories for raft Mu2, n=999 particles for each diffusivity coefficient.

*
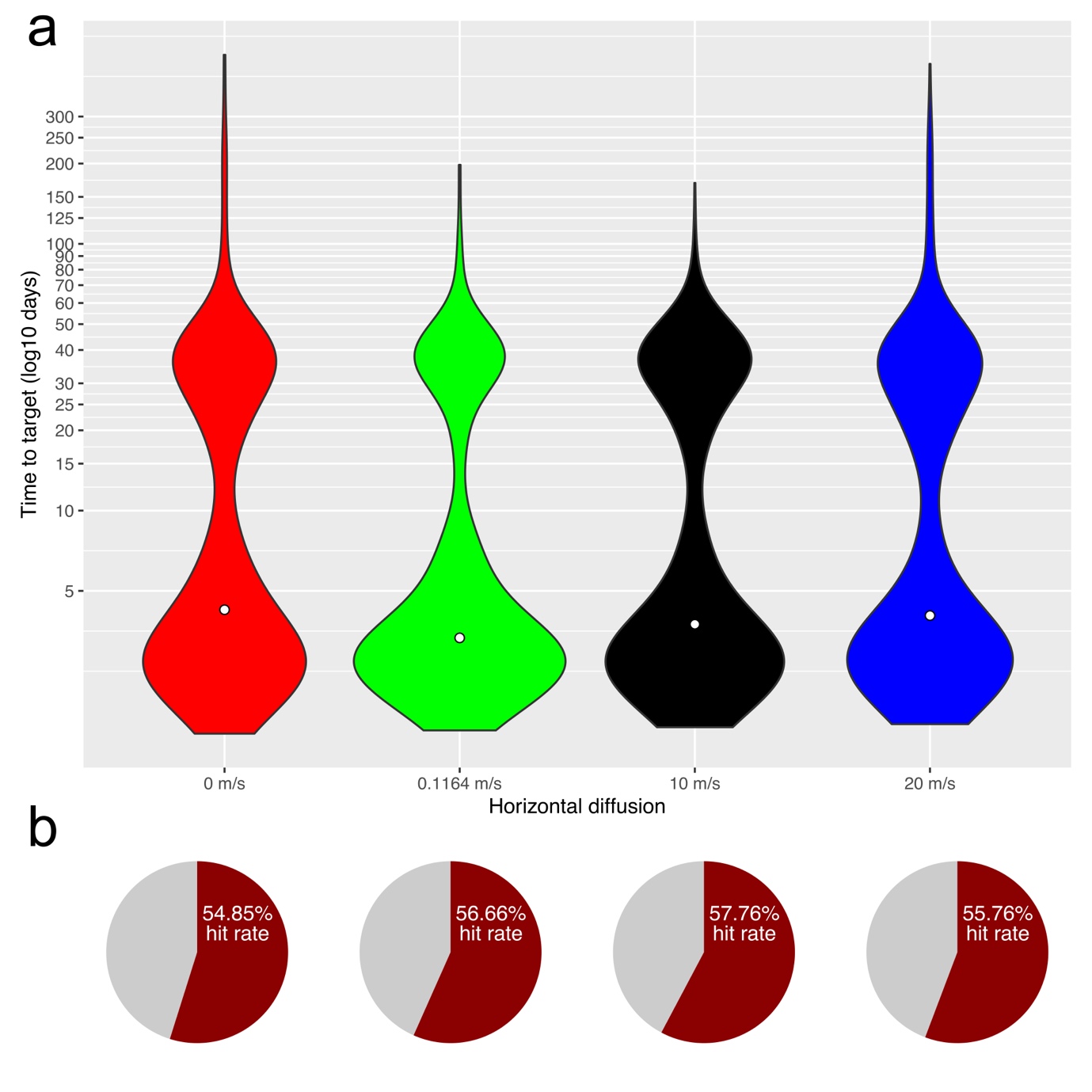
*

**Supplementary Figure S9** – a) Effects of horizontal diffusivity coefficient in Open Drift on time taken for raft Mu2 to reach source location, and b) proportion of particles reaching source/target zone. 999 particles were used for each diffusivity coefficient.
