# Supplementary material for: Host dispersal relaxes selective pressures in rafting microbiomes and triggers successional changes
Source: Nat Commun. 2024 Dec 30;15:10759. doi: 10.1038/s41467-024-54954-z (PMC11685921; doi:10.1038/s41467-024-54954-z)
Supplement: Supplementary file 3 — Reporting Summary [file 41467_2024_54954_MOESM3_ESM.pdf]

nature portfolio

Corresponding author(s): William S Pearman

Last updated by author(s): 10/21/2024

Reporting Summary

Nature Portfolio wishes to improve the reproducibility of the work that we publish. This form provides structure for consistency and transparency in reporting. For further information on Nature Portfolio policies, see our Editorial Policies and the Editorial Policy Checklist.

Statistics

For all statistical analyses, confirm that the following items are present in the figure legend, table legend, main text, or Methods section.

n/a	Confirmed	
☐	☒	The exact sample size (*n*) for each experimental group/condition, given as a discrete number and unit of measurement
☐	☒	A statement on whether measurements were taken from distinct samples or whether the same sample was measured repeatedly
☐	☒	The statistical test(s) used AND whether they are one- or two-sided<br>*Only common tests should be described solely by name; describe more complex techniques in the Methods section.*
☐	☒	A description of all covariates tested
☒	☐	A description of any assumptions or corrections, such as tests of normality and adjustment for multiple comparisons
☐	☒	A full description of the statistical parameters including central tendency (e.g. means) or other basic estimates (e.g. regression coefficient) AND variation (e.g. standard deviation) or associated estimates of uncertainty (e.g. confidence intervals)
☐	☒	For null hypothesis testing, the test statistic (e.g. *F*, *t*, *r*) with confidence intervals, effect sizes, degrees of freedom and *P* value noted<br>*Give P values as exact values whenever suitable.*
☒	☐	For Bayesian analysis, information on the choice of priors and Markov chain Monte Carlo settings
☒	☐	For hierarchical and complex designs, identification of the appropriate level for tests and full reporting of outcomes
☒	☐	Estimates of effect sizes (e.g. Cohen's *d*, Pearson's *r*), indicating how they were calculated

Our web collection on statistics for biologists contains articles on many of the points above.

Software and code

Policy information about availability of computer code

Data collection	We used OpenDrift1.10.7 under Python 3.10.12 to create modelled trajectories for our rafting kelp samples.
Data analysis	We used R4.2.1, OpenDrift1.10.7 under Python 3.10.12, IQ-TREEv 2.2, and STACKs (v.2.58) as principal software for our data analysis. Analysis code is available through https://github.com/wpearman1996/kelp_rafting_MS and https://doi.org/10.5281/zenodo.13910085.

For manuscripts utilizing custom algorithms or software that are central to the research but not yet described in published literature, software must be made available to editors and reviewers. We strongly encourage code deposition in a community repository (e.g. GitHub). See the Nature Portfolio guidelines for submitting code & software for further information.

Data

Policy information about availability of data

All manuscripts must include a data availability statement. This statement should provide the following information, where applicable:

- Accession codes, unique identifiers, or web links for publicly available datasets
- A description of any restrictions on data availability
- For clinical datasets or third party data, please ensure that the statement adheres to our policy

All genetic data underlying this paper has been deposited in the Aotearoa Genomics Data Repository - https://doi.org/10.57748/m4fe-0d07 and https://doi.org/10.57748/RDXN-1598 all other data is derived from these, and where possible (i.e., lat and longs of rafts) have been deposited in our GitHub - https://github.com/wpearman1996/kelp_rafting_MS and https://doi.org/10.5281/zenodo.13910085.

Research involving human participants, their data, or biological material

Policy information about studies with human participants or human data. See also policy information about sex, gender (identity/presentation), and sexual orientation and race, ethnicity and racism.

Reporting on sex and gender	*Use the terms sex (biological attribute) and gender (shaped by social and cultural circumstances) carefully in order to avoid confusing both terms. Indicate if findings apply to only one sex or gender; describe whether sex and gender were considered in study design; whether sex and/or gender was determined based on self-reporting or assigned and methods used. Provide in the source data disaggregated sex and gender data, where this information has been collected, and if consent has been obtained for sharing of individual-level data; provide overall numbers in this Reporting Summary. Please state if this information has not been collected. Report sex- and gender-based analyses where performed, justify reasons for lack of sex- and gender-based analysis.*
Reporting on race, ethnicity, or other socially relevant groupings	*Please specify the socially constructed or socially relevant categorization variable(s) used in your manuscript and explain why they were used. Please note that such variables should not be used as proxies for other socially constructed/relevant variables (for example, race or ethnicity should not be used as a proxy for socioeconomic status). Provide clear definitions of the relevant terms used, how they were provided (by the participants/respondents, the researchers, or third parties), and the method(s) used to classify people into the different categories (e.g. self-report, census or administrative data, social media data, etc.) Please provide details about how you controlled for confounding variables in your analyses.*
Population characteristics	*Describe the covariate-relevant population characteristics of the human research participants (e.g. age, genotypic information, past and current diagnosis and treatment categories). If you filled out the behavioural & social sciences study design questions and have nothing to add here, write "See above."*
Recruitment	*Describe how participants were recruited. Outline any potential self-selection bias or other biases that may be present and how these are likely to impact results.*
Ethics oversight	*Identify the organization(s) that approved the study protocol.*

Note that full information on the approval of the study protocol must also be provided in the manuscript.

Field-specific reporting

Please select the one below that is the best fit for your research. If you are not sure, read the appropriate sections before making your selection.

☐ Life sciences ☐ Behavioural & social sciences ☒ Ecological, evolutionary & environmental sciences

For a reference copy of the document with all sections, see nature.com/documents/nr-reporting-summary-flat.pdf

Ecological, evolutionary & environmental sciences study design

All studies must disclose on these points even when the disclosure is negative.

Study description	We examined the effects of long-distance dispersal of hosts on their associated microbiomes by comparing microbiomes of rafting kelp to those of non-rafting kelp. Principally we used a factorial design, and considered the effects & interactions of environmental temperature/variability, dispersal period, rafting status, and host genetics on microbiome structure. We had 37 kelp rafts collected, for which we have triplicate microbiome swabs analyzed - these were compared to data from another paper which examined the microbiomes of non-rafting kelp (https://doi.org/10.1093/aob/mcad151)
Research sample	Microbiome and genetic samples from sets of rafting kelp (n=37), and of non-rafting kelp (n=78) from a previous study (https://doi.org/10.1093/aob/mcad151)
Sampling strategy	For rafts - samples were collected opportunistically, wherever possible - thus we were constrained by the abundance of kelp rafts. Samples from previous research were used for non-rafts and were constrained by that research.
Data collection	Samples were collected by rinsing kelp first with artificial seawater and then microbial swabs were collected from rinsed tissue. Tissue clippings were also collected. At each point, negative microbial controls were also collected. Lead author William Pearman collected and recorded the data on a mobile phone.
Timing and spatial scale	Non-rafts were collected from around New Zealand in early 2022, while raft samples were opportunistically collected across a well-established and studied marine transect (the munida) off the coast of New Zealand every 2 months for two years. Non-raft data were collected in January 2022, while raft data were collected at 2 monthly intervals starting on 30/1/2021 through to 25/3/2022 - samples were collected from a 60km transect off shore of Dunedin.
Data exclusions	Two rafts samples were excluded from environmental analyses which relied on assessment of source location - as these samples could not be confidently assigned to source locations due to inconclusive data.
Reproducibility	We verified our opendrift trajectories by validating them against existing open datasets of drifters to ensure we were producing comparable data to known quantitative data on rafts/drifters. Effect sizes were calculated for this analysis.

Randomization	Samples were randomly assigned into groups for both DNA extractions and positions on plates for sequencing. Blinding was not otherwise used.
Blinding	Blinding was not used as the principal goal was to identify the drivers of community assembly between rafts and non-rafts.

Did the study involve field work? ☒ Yes ☐ No

Field work, collection and transport

Field conditions	Intertidal field work was conducted 2 hours prior to low tide, within the same 2 week period. for rafts - samples were collected opportunistically during suitable boating weather.
Location	Around New Zealand, and off shore otago - lats and longs provided within the manuscript supplementary material. These data vary for each raft sample - as such have not been included in this form, due the large nature of such data.
Access & import/export	Samples were collected within New Zealand, and followed the permitting requirements by acquisition of national permits. Permit number - 824-2 and 644 from the Ministry of Primary Industries
Disturbance	No disturbances

Reporting for specific materials, systems and methods

We require information from authors about some types of materials, experimental systems and methods used in many studies. Here, indicate whether each material, system or method listed is relevant to your study. If you are not sure if a list item applies to your research, read the appropriate section before selecting a response.

Materials & experimental systems

n/a	Involved in the study
☒	☐ Antibodies
☒	☐ Eukaryotic cell lines
☒	☐ Palaeontology and archaeology
☒	☐ Animals and other organisms
☒	☐ Clinical data
☒	☐ Dual use research of concern
☒	☐ Plants

Methods

n/a	Involved in the study
☒	☐ ChIP-seq
☒	☐ Flow cytometry
☒	☐ MRI-based neuroimaging

Plants

Seed stocks	*Report on the source of all seed stocks or other plant material used. If applicable, state the seed stock centre and catalogue number. If plant specimens were collected from the field, describe the collection location, date and sampling procedures.*
Novel plant genotypes	*Describe the methods by which all novel plant genotypes were produced. This includes those generated by transgenic approaches, gene editing, chemical/radiation-based mutagenesis and hybridization. For transgenic lines, describe the transformation method, the number of independent lines analyzed and the generation upon which experiments were performed. For gene-edited lines, describe the editor used, the endogenous sequence targeted for editing, the targeting guide RNA sequence (if applicable) and how the editor was applied.*
Authentication	*Describe any authentication procedures for each seed stock used or novel genotype generated. Describe any experiments used to assess the effect of a mutation and, where applicable, how potential secondary effects (e.g. second site T-DNA insertions, mosiacism, off-target gene editing) were examined.*
